# DW-ReID: Vision–Language Learning for Person Re-Identification Under Diverse Weather Conditions

**DOI:** 10.3390/s26072263

**Published:** 2026-04-06

**Authors:** Lei Cai, Yuying Liang, Bin Wang, Hexi Li, Jinquan Yang, Tao Zhu

**Affiliations:** 1 College of Engineering, Huaqiao University, Quanzhou 362021, China; lcai_gxy@hqu.edu.cn; 2 School of Computing, Guangzhou College of Applied Science and Technology, Zhaoqing 526072, China; jmlihexi@163.com (H.L.); jinquanyang2023@163.com (J.Y.); 3 School of Computer Science and Cyber Engineering, Guangzhou University, Guangzhou 510006, China; dvl.wangbin@e.gzhu.edu.cn; 4 School of Electronic Engineering and Automation, Guilin University of Electronic Technology, Guilin 541004, China; zt21@guet.edu.cn

**Keywords:** person re-identification, adverse weather, vision–language learning

## Abstract

Person re-identification (ReID) under diverse weather conditions remains a critical yet insufficiently explored problem. Most existing ReID approaches are developed and benchmarked on clear-weather datasets, resulting in significant performance degradation when deployed in rainy, snowy, or hazy environments. Conventional image restoration methods, typically optimized for low-level image quality metrics, are often misaligned with the objectives of high-level identity discrimination and thus fail to improve the person ReID performance. To address these limitations, we propose DW-ReID, a *unified* framework that integrates weather-degraded image restoration with person re-identification tasks. The proposed DW-ReID is built upon a large-scale *Contrastive Language-Image Pre-training* (CLIP) model and achieved by a two-stage training paradigm. In the first stage, a set of learnable text prompts is optimized to construct identity-specific ambiguous descriptions for each person’s identity. In the second stage, the optimized text descriptions, together with a frozen text encoder, provide *language supervision* to jointly train a weather encoder, an image restorer, and a ReID encoder in an end-to-end manner. The experimental results on two our contributed synthetic datasets consistently demonstrate the effectiveness and superior performance of the proposed DW-ReID method.

## 1. Introduction

Person re-identification (ReID) is a fundamental computer vision task that aims to match and track persons across non-overlapping camera views, with broad applications in intelligent video surveillance [1,2,3], intelligent transportation systems [4], and more. Although substantial progress has been achieved, most existing ReID methods are developed, trained, and evaluated under clear-weather conditions using large-scale, high-quality datasets. Consequently, even state-of-the-art approaches will suffer significant performance degradation in adverse weather conditions, where visual degradations pose critical challenges for robust person ReID [5].

To prevent performance degradation in adverse weather conditions, several high-level computer vision studies incorporate image restoration algorithms as a pre-processing step to restore clear images. However, existing image restoration methods (e.g., de-raining [6,7,8,9], de-snowing [10,11,12], and de-hazing [13,14]) are not well suited for person ReID in adverse weather conditions and may even degrade performance in certain cases. This issue arises for two primary reasons [15]. First, they are typically optimized for low-level image quality metrics, such as PSNR and SSIM, which are weakly correlated with the objectives of person ReID. As a result, the restored image details may be irrelevant or even detrimental to high-level ReID models. Second, most existing image restoration models are typically designed to handle a single type of weather degradation, without considering the coexistence or variability of multiple adverse weather conditions.

To conduct person re-identification in adverse weather conditions, several approaches [5,16] have been developed. However, to our knowledge, current methods are typically tailored to specific weather conditions, lacking the generality required for outdoor person ReID, which must operate stably across diverse weather conditions. Thus, this inspired us to design a *unified* framework that integrates low-level weather-degraded image restoration and high-level person ReID tasks. Specifically, in this paper, we propose DW-ReID to explore vision–language learning for person ReID under diverse weather conditions, such as clear, rainy, hazy, and snowy environments. The proposed DW-ReID method is built upon pre-trained CLIP and achieved by a two-stage training paradigm. In the first stage, a series of learnable text descriptions is optimized to construct identity-specific ambiguous text descriptions for each person’s identity (ID). In the second stage, these optimized text descriptions, together with a frozen text encoder, provide *language supervision* to jointly train a weather encoder, an image restorer, and a ReID encoder. The joint training in the second stage is an end-to-end manner, which tightly integrates the low-level weather-degraded image restoration and the high-level person ReID tasks into a unified framework.

In summary, the main contributions of this article are as follows:

- We propose a unified framework DW-ReID to explore vision–language learning for person ReID under diverse weather conditions. The proposed DW-ReID can effectively reduce the interference of diverse adverse weather on person ReID.
- We introduce the two-stage training paradigm to optimize the proposed DW-ReID. Such a training paradigm enables the proposed method to obtain language supervision from pre-trained CLIP to handle the person ReID under diverse weather conditions.
- We contribute two synthetic weather-degraded image datasets for studying person ReID under multiple weather conditions, such as clear, rainy, snowy, and hazy environments. The experimental results consistently demonstrate the effectiveness and superiority of our method compared to other ReID approaches.

## 2. Related Work

### 2.1. Person Re-Identification

Person re-identification (ReID) aims to retrieve images of the same person’s identity across non-overlapping camera views. Early approaches [17,18,19,20] primarily relied on handcrafted feature extraction combined with metric learning techniques. With the advent of deep learning, research attention has gradually shifted toward representation learning based on deep neural networks [21]. A prevalent paradigm formulates task-specific loss functions to supervise the training of well-designed CNN backbones, such as ResNet [22], for learning discriminative identity-specific features. However, as person ReID is a fine-grained recognition task characterized by large intra-class variations and small inter-class differences [23], conventional CNN backbones often struggle to learn sufficiently discriminative features for reliably distinguishing visually similar identities. To enhance feature discriminability, some works [24,25,26] incorporate various effective mechanisms into CNN backbones to better mine identity-specific cues and reinforce weakly discriminative features. Beyond CNN-based person ReID approaches, a growing line of research [23,27,28,29,30] has adopted Vision Transformers (ViTs) [31] to leverage their global representation modeling capability for learning more robust and discriminative features from large-scale image datasets.

Although existing person ReID methods have demonstrated impressive performance, such success largely stems from the fact that they are predominantly trained and tested under consistent data distribution, since the images in the training and testing sets are commonly collected from clear weather conditions. However, when they are deployed across diverse weather conditions, these models often experience significant performance degradation due to pronounced distribution shifts induced by adverse weather. Considering this, Pang et al. [5] tackled the domain adaptation challenges in person ReID under hazy weather conditions. Their approach adopts a teacher–student architecture to learn haze-invariant representations under the supervision of an intrinsic similarity matrix, and further introduces an auxiliary discriminator to align the feature distribution of hazy images with that of clear images. In addition, they constructed two synthetic hazy ReID datasets to mitigate performance degradation when transferring the trained model to target domains affected by haze. Their method is specifically designed for specific hazy weather, which limits their generalization to other adverse weather conditions. To the best of our knowledge, there has not an effective method that can effectively perform person ReID under diverse weather conditions, such as clear, rainy, snowy, and hazy environments.

### 2.2. Vision–Language Learning

The large-scale Contrastive Language-Image Pre-training (CLIP) model [32] leverages extensive image–text datasets to pre-train paired image and text encoders through contrastive image–text alignment. Built upon the success of CLIP, a growing number of studies [33,34,35] have sought to enhance vision–language pre-training models, aiming to stimulate their potential for person ReID across diverse downstream tasks. To this end, Zhou et al. [33] proposed Context Optimization (CoOp), which learns data-specific text prompts for image classification. Subsequently, they extended this approach in [34] with Conditional Context Optimization (CoCoOp), wherein a lightweight meta-network generates a dynamic meta token for each image, allowing for prompt adaptation at the instance level rather than relying on static prompts as in CoOp. Instead of learning text prompts, Gao et al. [35] introduced CLIP-Adapter, which fine-tunes both image and text encoders using feature adapters, providing an alternative strategy to enhance CLIP for downstream tasks.

Since CLIP [32] has demonstrated a remarkable capacity to capture rich semantic information from both images and text; this inspired researchers to explore their potential for person ReID [36,37,38,39,40,41]. For example, Li et al. [36] introduced CLIP-ReID, a pioneering approach that adapts vision–language pre-trained models to the ReID task. Specifically, CLIP-ReID leverages a pre-trained CLIP model to constrain the image encoder via language descriptions generated by the text encoder. Building on this, Wang et al. [37] integrated self-supervision into large-scale vision–language pre-trained models (CLIP) for image re-identification. Lin et al. [38] proposed enhancing fine-grained visual features through part-informed language supervision. Specifically, they combined identity labels with parsing maps to generate pixel-level text prompts and fused multi-stage visual features using a lightweight auxiliary head to achieve more precise image–text alignment. More recently, UniPT [39] and CFine [40] applied the CLIP to text-to-image person ReID. However, both approaches rely on extensive manual annotation of text labels, and the generation of high-quality prompts remains a labor-intensive task requiring substantial domain expertise.

## 3. Methodology

**Figure 1:**
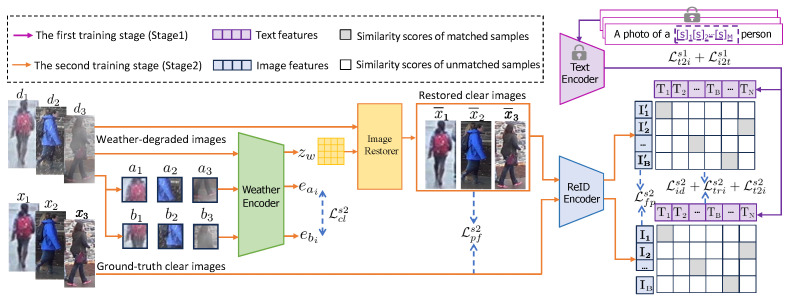
Schematic illustration of our proposed DW-ReID, which contains two training stages. In the first stage (purple arrows), the parameters in the text encoder and ReID encoder are frozen, and a set of learnable text tokens ${\left[s\right]}_{1}{\left[s\right]}_{2}\ldots {\left[s\right]}_{M}$ is optimized to construct identity-specific ambiguous text descriptions for each person’s identity. In the second stage (orange arrows), the text encoder and the optimized text descriptions in the first stage are kept frozen, while the parameters in the weather encoder, image restorer, and ReID encoder are optimized jointly in an end-to-end manner with *language supervision* imposed by text descriptions and the text encoder.

The main objective of this work is to present a *unified* method to handle person re-identification under diverse weather conditions. For that, we propose DW-ReID, which integrates the weather-degraded image restoration with person re-identification. The schematic illustration of the proposed DW-ReID is shown in Figure 1, which consists of four components: *weather encoder*, *image restorer*, *text encoder*, and *ReID encoder*. To be adapted to the person ReID, the proposed DW-ReID is trained in a two-stage paradigm, as follows.

### 3.1. The First Training Stage

The first training stage focuses on learning ambiguous text descriptions for each person’s identity. Specifically, following prior practices [36,37], we introduce identity-specific learnable tokens to construct identity dependent ambiguous text descriptions, which are optimized independently for each person’s identity. As shown in Figure 1, the text description fed into the text encoder is formulated as “A photo of a ${\left[s\right]}_{1}{\left[s\right]}_{2}\ldots {\left[s\right]}_{M}$ person”, where each ${\left[s\right]}_{m}$ (m∈{1,…,M}) denotes a learnable text token embedded in the same space as word embeddings, and *M* is the number of such tokens. Subsequently, the text description and the corresponding ground-truth clear image are independently encoded by the text encoder and the ReID encoder, yielding the text feature embedding *T* and the image feature embedding *I*, respectively. The image feature embedding *I* provides *vision supervision* for text token learning through a text-to-image contrastive loss $L_{t2i}^{s1}$ and an image-to-text contrastive loss $L_{i2t}^{s1}$. Notably, during this stage, the parameters of both the text encoder and the ReID encoder are kept fixed, while the learnable text tokens ${\left[s\right]}_{1}{\left[s\right]}_{2}\ldots {\left[s\right]}_{M}$ are optimized by calculating the contrastive losses $L_{t2i}^{s1}$ and $L_{i2t}^{s1}$, as follows:

(1) $$L_{t2i}^{s1}=-\frac{1}{\left|P(y_{i})\right|}\sum_{p\in P(y_{i})}\log\frac{\exp(I_{x_{p}}\cdot T_{y_{i}})}{\sum_{v=1}^{B}\exp\left(I_{x_{i}}\cdot T_{y_{v}}\right)},$$

(2) $$L_{i2t}^{s1}=-\frac{1}{\left|P(x_{i})\right|}\sum_{p\in P(x_{i})}\log\frac{\exp(I_{x_{i}}\cdot T_{y_{p}})}{\sum_{v=1}^{B}\exp\left(I_{x_{v}}\cdot T_{y_{i}}\right)},$$

where $x_{i}$ and $y_{i}$ represent the *i*-th image and the text description in a batch. $P\left(y_{i}\right)=\left\{p\in \left\{1,\ldots ,B\right\}:y_{p}=y_{i}\right\}$ and $P\left(x_{i}\right)=\left\{p\in \left\{1,\ldots ,B\right\}:x_{p}=x_{i}\right\}$ are the sets of all positives with the same identity, *B* is the batch size, and |·| represents the cardinality of $P(y_{i})$. $T_{y_{i}}$ denotes the text feature embedding corresponding to text description $y_{i}$, and $I_{x_{i}}$ denotes the image feature embedding corresponding to image sample $x_{i}$.

Ultimately, by minimizing the joint contrastive loss $L_{t2i}^{s1}$ and $L_{i2t}^{s1}$, gradients are back-propagated through the frozen text encoder to update the learnable text tokens ${\left[s\right]}_{1}{\left[s\right]}_{2}\ldots {\left[s\right]}_{M}$:

(3) $$\begin{array}{c} L^{s1}=L_{t2i}^{s1}+L_{i2t}^{s1}. \end{array}$$

### 3.2. The Second Training Stage

In this stage, the text encoder and the optimized text descriptions obtained in the first stage are kept frozen, while the weather encoder, image restorer, and ReID encoder are trained jointly in an end-to-end manner. Among them, the weather encoder is designed to learn weather-aware degradation representation $z_{w}$ to distinguish different weather types. Specifically, a set of weather-degraded images {$d_{1},d_{2},d_{3}$}, corresponding to rain, snow, and haze, are randomly cropped to obtain two sets of image patches {$a_{1},a_{2},a_{3}$} and {$b_{1},b_{2},b_{3}$}. These image patches are then passed through the weather encoder to obtain the corresponding weather-aware feature embeddings $e_{a}$ and $e_{b}$. To regularize the training of the weather encoder, a contrastive learning loss $L_{cl}^{s2}$ is formulated:

(4) $$L_{cl}^{s2}=\sum_{i=1}^{3}-\log\frac{\exp(\left(e_{a_{i}}\cdot e_{b_{i}}\right)/T)}{\sum_{j=1}^{N_{queue}}\exp\left(\left(e_{a_{i}}\cdot e_{j}\right)/T\right)},$$

where $a_{i}$ and $b_{i}$ are the positive samples because they represent the same weather, whereas $e_{a_{i}}$ and $e_{b_{i}}$ are their corresponding feature embeddings. $e_{j}$ denotes the feature embeddings of negative samples relative to $e_{a_{i}}$, and T is a temperature coefficient. To overcome the limited number and insufficient diversity of negatives in a mini-batch, we introduce a MoCo-style FIFO memory bank as the negative sample queue to maintain a group of feature embeddings of negative samples during training. $N_{\mathrm{queue}}$ represents the storage size in the queue. In each training iteration, the feature embeddings of negative samples in the queue are updated according to the first-in, first-out principle. During training, the contrastive learning loss $L_{\mathrm{cl}}$ is minimized to reduce the feature distribution distance among matched positive pairs such as $e_{a_{i}}$ and $e_{b_{i}}$, while increasing the feature distribution distance among non-matched negative pairs such as $e_{a_{i}}$ and $e_{j}$. Thus, $L_{\mathrm{cl}}$ encourages the weather encoder to focus on learning weather-aware degradation representation $z_{w}$ from the input weather-degraded images.

The image restorer is designed to restore clear person images {${\bar{x}}_{1},{\bar{x}}_{2},{\bar{x}}_{3}$} from their corresponding weather-degraded images {$d_{1},d_{2},d_{3}$}. The restoration process is guided by the weather-aware degradation representation $z_{w}$ learned from the weather encoder, aiming to achieve *all-in-one* weather-degraded image restoration. Furthermore, to guarantee the fidelity of the restored images to their clear counterparts, a *pixel-wise fidelity loss* $L_{pf}^{s2}$ is introduced to regularize the training of the image restorer, as follows:

(5) $$L_{pf}^{s2}=\frac{1}{3}\sum_{i=1}^{3}{∥x_{i}-{\bar{x}}_{i}∥}_{1},$$

where $x_{i}$ denotes the ground-truth clear image, whereas ${\bar{x}}_{i}$ is the corresponding restored clear image. On the other hand, a *feature-wise perception loss*$L_{fp}^{s2}$ is also introduced to align the restored clear image ${\bar{x}}_{i}$ and its corresponding ground truth $x_{i}$ from an identity-perception perspective:

(6) $$L_{fp}^{s2}=\frac{1}{3}\sum_{i=1}^{3}{∥I_{i}-I_{i}^{\prime }∥}_{1},$$

where $I_{i}^{\prime }$ and $I_{i}$ represent the identity-specific features extracted from restored clear image ${\bar{x}}_{i}$ and its corresponding ground truth $x_{i}$ using the ReID encoder.

The ReID encoder is introduced to learn the descriminative identity-specific features for the final person ReID. To boost the ReID performance, we follow a widely used strong ReID training paradigm [36,37,42] in which a triplet loss $L_{tri}^{s2}$ and an identity loss $L_{id}^{s2}$ with label smoothing is applied to regularize the ReID encoder:

(7) $$L_{tri}^{s2}=\max\left(d_{p}^{\prime }-d_{n}^{\prime }+\alpha ,0\right)+\max\left(d_{p}-d_{n}+\alpha ,0\right),$$

(8) $$L_{id}^{s2}=\sum_{v=1}^{B}-q_{v}^{\prime }\log\left(p_{v}^{\prime }\right)+\sum_{v=1}^{B}-q_{v}\log\left(p_{v}\right),$$

where $d_{p}^{\prime }$ and $d_{n}^{\prime }$ represent the feature distances with respect to positive sample pairs and negative sample pairs for restored clear images, whereas $d_{p}$ and $d_{n}$ denote feature distances with respect to positive sample pairs and negative sample pairs for ground truth clear images, α is the margin of $L_{tri}^{s2}$. In addition, *B* denotes the batch size, whereas $q_{v}^{\prime }$ and $q_{v}$ represent values in the target distribution of restored and ground-truth clear images. $p_{v}^{\prime }$ and $p_{v}$ denote the identity prediction logits of class *v* for restored and ground-truth clear images. To fully exploit the advantage of pre-trained CLIP for each image, the text feature embeddings obtained in the first training stage is introduced to impose *language supervision* to the ReID encoder by calculating an image-to-text cross-entropy loss $L_{t2i}^{s2}$:

(9) $$L_{t2i}^{s2}=\sum_{v=1}^{B}-q_{v}^{\prime }\log\frac{\exp(I_{i}^{\prime }\cdot T_{y_{v}})}{\sum_{j=1}^{N}\exp\left(I_{i}^{\prime }\cdot T_{y_{j}}\right)}+\sum_{v=1}^{B}-q_{v}\log\frac{\exp(I_{i}\cdot T_{y_{v}})}{\sum_{j=1}^{N}\exp\left(I_{i}\cdot T_{y_{j}}\right)}.$$

Ultimately, the weather encoder, image restorer, and ReID encoder are trained jointly in an end-to-end manner. And the final loss function used in the second stage is expressed as follows:

(10) $$L^{s2}=\lambda_{cl}L_{cl}^{s2}+\lambda_{pf}L_{pf}^{s2}+\lambda_{fp}L_{fp}^{s2}+\lambda_{tri}L_{tri}^{s2}+\lambda_{id}L_{id}^{s2}+\lambda_{t2i}L_{t2i}^{s2},$$

where $\lambda_{cl}$, $\lambda_{pf}$, $\lambda_{fp}$, $\lambda_{tri}$, $\lambda_{id}$, and $\lambda_{t2i}$ denote the corresponding weighting parameters. The two-stage training procedure of DW-ReID is summarized in Algorithm 1.
**Algorithm 1** The two-stage training procedure of DW-ReID**Input:** batch of images $\left\{d_{i},x_{i}\right\}$ and their corresponding texts $y_{i}$.**Parameter:** a set of learnable text tokens ${\left[s\right]}_{m}$ (m∈1,…,M) for all identities existing in training set, a weather encoder W(·), an image restorer R(·), a text encoder T(·), and a ReID encoder E(·), linear layers $f_{V}\left(\cdot \right)$ and $f_{T}\left(\cdot \right)$, a negative sample queue Q(·).1: Initialize T(·), E(·), $f_{V}\left(\cdot \right)$ and $f_{T}\left(\cdot \right)$ from the pre-trained CLIP. Initialize W(·), R(·), and ${\left[s\right]}_{m}$ (m∈1,…,M) randomly.2: **while** in the 1st stage **do**3:        $I_{x_{i}}=f_{V}\left(E\left(x_{i}\right)\right)$, $T_{y_{i}}=f_{T}\left(T\left(y_{i}\right)\right)$4:        Optimize ${\left[s\right]}_{m}$ by Equation (3)5: **end while**6: **while** in the 2nd stage **do**7:        $I_{x_{i}}=f_{V}\left(E\left(x_{i}\right)\right)$, $T_{y_{i}}=f_{T}\left(T\left(y_{i}\right)\right)$, $e_{a_{i}}=W\left(a_{i}\right)$8:        $e_{b_{i}}=W\left(b_{i}\right)$, $I_{i}=E\left(x_{i}\right)$, ${\bar{x}}_{i}=R\left(x_{i}\right)$, $I_{i}^{\prime }=E\left({\bar{x}}_{i}\right)$9:        Jointly optimize W(·), R(·), and E(·) by Equation (10)10:      Update the queue Q(·)11: **end while**

### 3.3. Network Architectures

**Figure 2:**
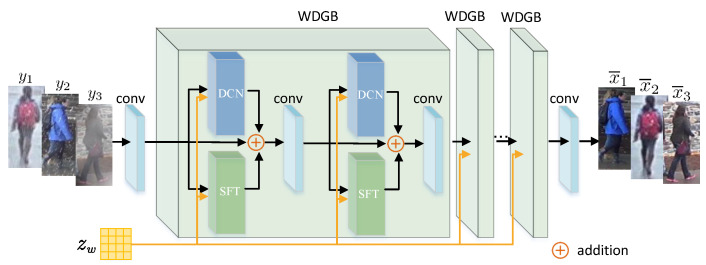
Schematic illustration of the image restorer, which takes the weather-degraded images {${\bar{y}}_{1},{\bar{y}}_{2},{\bar{y}}_{3}$} as input and is then sequentially fed them into a convolutional layer, several Weather-Degradation-Guided Blocks (WDGBs), and another convolutional layer to restore the clear person images {${\bar{x}}_{1},{\bar{x}}_{2},{\bar{x}}_{3}$}.

For the text encoder and the ReID encoder in our proposed DW-ReID, we adopt the CLIP pre-trained ViT-B/16 model [38] as the backbone. ViT-B/16 consists of 12 Transformer layers with a hidden dimension of 768. To be compatible with the output dimension of the text encoder, the identity-specific features produced by the ReID encoder are reduced from 768 to 512 dimensions via a linear layer. For the weather encoder, we employ a six-layer convolutional network to extract the weather-aware degradation representation $z_{w}$. For the image restorer, its architecture is illustrated in Figure 2, which contains several Weather-Degradation-Guided Block (WDGB) cascaded together. In each WDGB block, the weather-aware degradation representation $z_{w}$ is injected into a Deformable Convolution (DCN) [43] and a Spatial Feature Transform (SFT) [44]. With the guidance of $z_{w}$, DCN is able to adaptively adjust the receptive field to capture the rich texture information in the input weather-degraded images, while SFT is capable of adjusting the latent distribution of different weather degradations, thereby alleviating the feature distribution discrepancy between the restored clear images and the ground-truth clear ones.

## 4. Experiments

### 4.1. Synthetic Weather-Degraded Dataset and Evaluation Metrics

To achieve person ReID under diverse weather conditions, it is essential to construct a benchmark dataset containing person images captured under clear, rainy, snowy, and hazy environments. Nevertheless, collecting and annotating large-scale ReID datasets under diverse weather conditions remains prohibitively expensive and labor-intensive.

**Figure 3:**
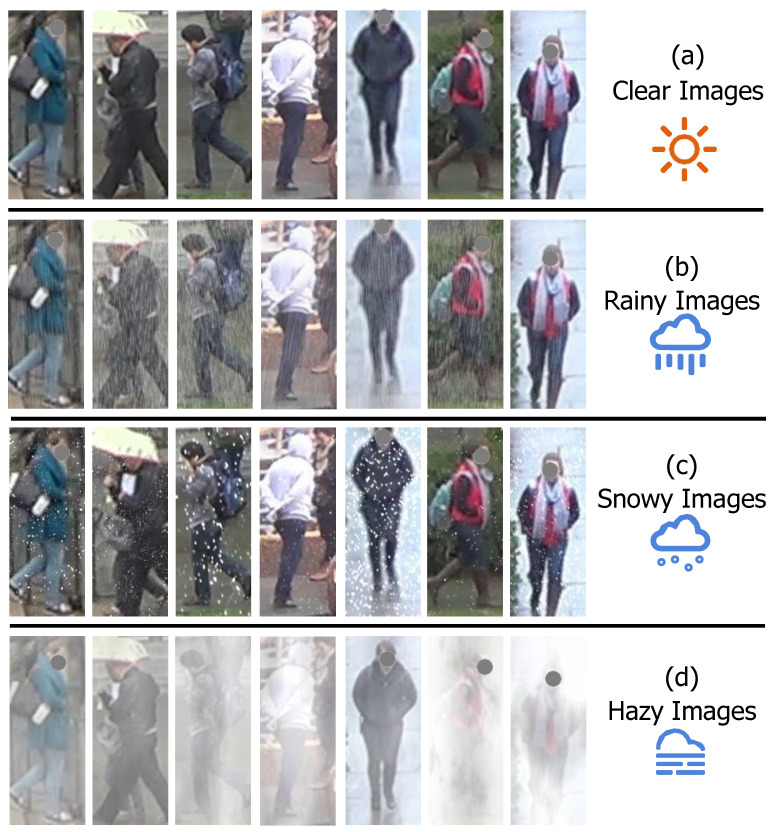
Some examples of our synthetic weather-degraded images in our DW-Market-1501 and DW-DukeMTMC-reID datasets, including clear images, rainy images, snowy images, and hazy images.

Considering that prior research has not yet involved person ReID under diverse weather conditions, this motivates us to synthesize weather-degraded images to reduce the negative impact of weather interference on ReID performance when deploying the models on real-world application scenarios. Specifically, we adopted the Imgaug library [45] to simulate degradations with respect to rain streaks, snowflakes, and haze. Then, we added these degradations on existing person images in Market-1501 [46] and DukeMTMC-reID [47] datasets so as to form two new datasets, termed DW-Market-1501 and DW-DukeMTMC-reID. For simulating the rain streaks, we set the degradation factors between 0.01 and 0.02, while controlling speed factors between 0.02 and 0.04. For simulating the snowflakes, we set the size of snowflakes between 0.1 and 0.7 while controlling speed factors between 0.007 and 0.03. For simulating the haze, we set the mean intensity between 220 and 255. Figure 3 showcases some examples of synthetic weather-degraded images in our DW-Market-1501 and DW-DukeMTMC-reID datasets. From Figure 3, we can observe that these images exhibit different degrees of degradation caused by rain, snow, and haze. More details about the DW-Market-1501 and DW-DukeMTMC-reID datasets are summarized in Table 1. For performance evaluation, we adopted the *Cumulative Matching Characteristics* (CMC) at Rank-1 and the *mean Average Precision* (mAP) as evaluation metrics.

### 4.2. Implementation Details

The proposed DW-ReID was implemented using the PyTorch (v1.13.1) deep learning framework on a single GPU of NVIDIA RTX A6000. In the first training stage, the Adam optimizer was applied to optimize the text tokens. At this stage, the model was trained for 120 epochs with an initial learning rate of $3.5\times 10^{-4}$. The learning rate was then decayed according to a cosine schedule. In the second stage, the weather encoder, image restorer, and ReID encoder were trained jointly in an end-to-end manner for 500 epochs. At this stage, the learning rate was initialized by $5\times 10^{-6}$ and linearly decayed by a factor of 0.5 for every 100 epochs. During the training procedure, we randomly sampled the person images with a batch size of 64 and then resized them to 256×128 resolutions. Afterwards, these resized images were further randomly cropped to 128×128 resolutions to be fed into the weather encoder. We fixed the weighting parameters of $\lambda_{cl}$, $\lambda_{pf}$, $\lambda_{fp}$, $\lambda_{tri}$, and $\lambda_{t2i}$ at 1.0, whereas $\lambda_{id}$ was fixed at 0.25.

### 4.3. Comparison with the State of the Arts

To conduct a comprehensive evaluation, we compare the proposed DW-ReID with several recently developed person ReID approaches. For a fair comparison, only the weather-degraded images (i.e., rain, snow, and haze) in the DW-Market-1501 and DW-DukeMTMC-reID datasets were processed using Histoformer [48] to obtain clear versions, while the images that were already captured under clear-weather conditions were left unchanged and directly used. Specifically, for both the training and testing sets, the degraded images were first restored to their clear counterparts using Histoformer [48]. These restored images were then combined with the original clear images to form corresponding training and testing sets, and all competing ReID models were subsequently retrained and evaluated on these sets. This protocol was designed to ensure fairness by avoiding any artificial modification of already clear images. The corresponding quantitative results are reported in Table 2 and Table 3, respectively. As can be seen, the proposed DW-ReID achieves the best performance on average under diverse weather conditions and also exceeds the second-best method CLIMB-ReID [49] in rainy, snowy, and hazy environments. For example, compared with the second-best ReID method SVLL-ReID [37], the proposed DW-ReID exceeds it by 3.0%/1.9% in terms of mAP/Rank-1 on DW-Market-1501 and by 1.8%/2.9% on DW-DukeMTMC-reID on the overall average performance evaluation. These results consistently demonstrate the effectiveness and superiority of our proposed DW-ReID in handling person re-identification under diverse weather conditions.

### 4.4. Ablation Study

To further validate the proposed DW-ReID method, we conducted deeper analyses on the DW-DukeMTMC-reID dataset. Specifically, we mainly investigate the following three aspects: (1) The effect of two-stage training for DW-ReID. (2) The necessity of joint training in the second stage. (3) The necessity of adverse weather removal for person ReID.

**The effect of two-stage training for DW-ReID:** The proposed DW-ReID contains two training stages to be adapted to person re-identification. The first training stage aims to learn ambiguous text descriptions for each person’s identity. Then, in the second training stage, the text encoder and the optimized text descriptions obtained in the first stage are fixed, while the weather encoder, image restorer, and the ReID encoder are trained jointly in an end-to-end manner. To investigate the effect of two-stage training for DW-ReID, we excluded the first training stage (i.e., without the optimization of text descriptions and does not impose language supervision to the ReID encoder), while directly conducting the second training stage for person ReID (i.e., conducting the joint training of the weather encoder, image restorer, and ReID encoder). The corresponding results are reported in Table 4. As can be seen, the mAP and Rank-1 resulted by two-stage training are significantly superior to those by solely performing the second training stage. This implies that two-stage training scheme can fully stimulate the potential of vision–language learning to enhance the ReID performance under diverse weather conditions.

**The necessity of joint training in the second stage:** In DW-ReID, the weather encoder and the image restorer constitute the image restoration part, while the ReID encoder constitutes the person ReID part. To gain insight into whether training these two parts separately or jointly in the second stage is more beneficial for pedestrian ReID, we further carried out an ablation experiment, and Table 5 documents the corresponding results. One can observe that the mAP and Rank-1 resulted by joint training consistently outperform those of separated training; this is due to the fact that joint training could tightly integrate the weather-degraded image restoration and the person identification tasks into a unified framework. This enables the low-level image restoration task to better assist the high-level person re-identification task across diverse weather conditions. This study fully demonstrates the necessity of joint training in the second stage.

**Figure 4:**
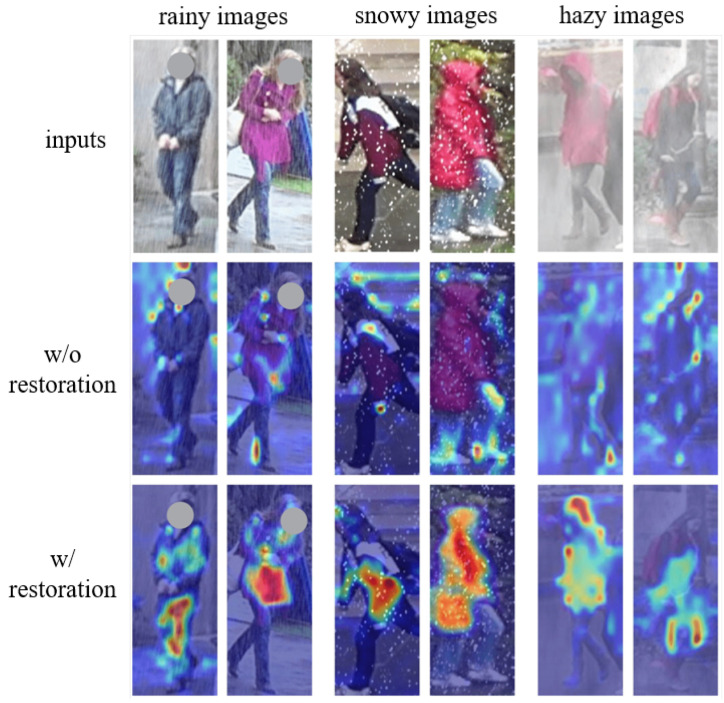
Qualitative comparison of attention maps with (w/) and without (w/o) conducting image restoration in DW-ReID.

**The necessity of adverse weather removal for person ReID:** In order to analyze the necessity of adverse weather removal for person ReID, we compared the re-identification performance with (w/) and without (w/o) image restoration in the proposed DW-ReID. Note that without image restoration means that we removed the weather encoder and image restorer in DW-ReID and directly used the ReID encoder for person re-identification. The corresponding quantitative results on the test set of DW-Market-1501 and DW-DukeMTMC-reID are documented in Table 6 and Table 7, respectively. One can see that, without image restoration, the performance of person re-identification drops dramatically compared to that with image restoration in the proposed DW-ReID. These quantitative results demonstrate the necessity of adverse weather removal for person ReID. In addition, to qualitatively demonstrate the necessity of adverse weather removal for person ReID, Figure 4 compares the attention maps with and without conducting image restoration in DW-ReID. One can see that, with image restoration, the proposed DW-ReID can focus more on the human body, which is beneficial for person retrieval. This benefit is attributed to the fact that the image restoration function in DW-ReID can effectively remove the interference of diverse adverse weather on person ReID.

### 4.5. Cross-Dataset Evaluation

To evaluate the generalization ability, a cross-dataset evaluation was carried out among the proposed DW-ReID and three recently-developed baselines, including CLIP-ReID [36], SVLL-ReID [37], and CLIMB-ReID [49]. In this cross-dataset evaluation, all of the compared methods are either trained on the DW-Market-1501 dataset and then tested on DW-DukeMTMC-reID, or vice versa, trained on DW-DukeMTMC-reID and then tested on DW-Market-1501.

The corresponding results are shown in Table 8 and Table 9, respectively. As can be seen, the performance of all the compared methods significantly declined in the cross-dataset evaluation compared to the performance documented in Table 2 and Table 3, respectively. This can be attributed to the fact that models trained on a specific dataset tend to learn dataset-specific characteristics, including particular distributions of pedestrian appearance, illumination conditions, viewpoints, and backgrounds. Consequently, when evaluated on a different dataset, their generalization capability is often limited, as the target dataset may present substantially different visual statistics and data distributions. Nevertheless, from Table 8 and Table 9, we can also observe that, in the cross-dataset evaluation, the proposed DW-ReID still outperforms other competing methods on the overall average performance. This cross-dataset evaluation demonstrates that the proposed DW-ReID method presents better or competitive generalization ability compared with the other three competing methods.

## 5. Conclusions

In this paper, we address the critical yet underexplored problem of person re-identification in multiple weather conditions. Specifically, we propose DW-ReID, an *all-in-one* learning framework that is built upon CLIP with a two-stage training paradigm. In the first stage, a set of learnable text descriptions is optimized to produce identity-specific ambiguous text descriptions for each person’s identity. In the second stage, those optimized text descriptions together with a frozen text encoder provide *language supervision* to jointly train a weather encoder, an image restorer, and a ReID encoder. The experimental results consistently demonstrate the effectiveness and superiority of our proposed DW-ReID method against other competing ReID approaches. 

## Figures and Tables

**Table 1:** Statistics of DW-Market-1501 and DW-DukeMTMC-reID datasets.

Dataset	Clear	Rainy	Snowy	Hazy	ID	Camera	Training	Gallery	Query
DW-Market-1501	36,036	36,036	36,036	36,036	1501	6	51,744	78,928	13,472
DW-DukeMTMC-reID	36,411	36,411	36,411	36,411	1404	8	66,088	70,644	8912

**Table 2:** Quantitative performance comparison of different person ReID methods on the testing set of DW-Market-1501.

Methods	Clear		Rainy		Snowy		Hazy		Average	
	mAP	Rank-1	mAP	Rank-1	mAP	Rank-1	mAP	Rank-1	mAP	Rank-1
DC-Former [23]	84.8	93.2	79.2	89.1	80.0	89.6	68.9	81.3	78.2	88.3
CLIP-ReID [36]	88.5	93.9	84.4	90.2	85.5	91.9	72.0	84.1	82.6	90.1
SVLL-ReID [37]	91.3	95.9	85.3	90.7	86.1	93.6	74.2	87.8	84.2	92.0
CLIMB-ReID [49]	**92.6**	**96.6**	86.0	91.2	88.3	95.0	75.5	89.1	85.6	93.0
**DW-ReID (Ours)**	90.7	95.6	**88.1**	**94.4**	**90.0**	**95.2**	**80.1**	**90.5**	**87.2**	**93.9**

**Table 3:** Quantitative performance comparison of different person ReID methods on the testing set of DW-DukeMTMC-reID.

Methods	Clear		Rainy		Snowy		Hazy		Average	
	mAP	Rank-1	mAP	Rank-1	mAP	Rank-1	mAP	Rank-1	mAP	Rank-1
DC-Former [23]	79.8	89.2	73.9	85.8	80.4	89.6	64.5	76.7	74.7	85.3
CLIP-ReID [36]	81.1	89.8	79.3	86.9	77.6	88.5	70.4	80.0	77.1	86.3
SVLL-ReID [37]	**84.4**	**91.7**	80.7	88.3	78.5	89.1	71.9	80.8	78.9	87.5
CLIMB-ReID [49]	78.5	88.4	76.5	87.9	77.7	88.2	69.5	84.3	75.6	87.2
**DW-ReID (Ours)**	83.4	91.4	**81.1**	**90.7**	**82.8**	**91.0**	**75.6**	**88.6**	**80.7**	**90.4**

**Table 4:** mAP and Rank-1 results with (✓) and without (×) employing two-stage training in the proposed DW-ReID method.

Two-Stage Training	Clear		Rain		Snow		Haze		Average	
	mAP	Rank-1	mAP	Rank-1	mAP	Rank-1	mAP	Rank-1	mAP	Rank-1
×	82.0	90.0	80.0	89.2	80.1	89.7	73.2	86.2	78.8	88.8
✓	**83.4**	**91.4**	**81.1**	**90.7**	**82.8**	**91.0**	**75.6**	**88.6**	**80.7**	**90.4**

**Table 5:** mAP and Rank-1 results for different training modes in the second stage.

Training Mode	Clear		Rain		Snow		Haze		Average	
	mAP	Rank-1	mAP	Rank-1	mAP	Rank-1	mAP	Rank-1	mAP	Rank-1
Separated	83.1	90.8	75.0	86.3	78.2	88.0	73.7	86.3	77.5	87.8
Joint	**83.4**	**91.4**	**81.1**	**90.7**	**82.8**	**91.0**	**75.6**	**88.6**	**80.7**	**90.4**

**Table 6:** mAP and Rank-1 results of the proposed DW-ReID with (w/) and without (w/o) image restoration on DW-Market-1501.

Method: DW-ReID	Clear		Rain		Snow		Haze		Average	
	mAP	Rank-1	mAP	Rank-1	mAP	Rank-1	mAP	Rank-1	mAP	Rank-1
w/o restoration	85.1	93.4	78.5	90.3	79.2	90.6	51.8	72.0	73.6	86.5
w/restoration	**90.7**	**95.6**	**88.1**	**94.4**	**90.0**	**95.2**	**80.1**	**90.5**	**87.2**	**93.9**

**Table 7:** mAP and Rank-1 results of the proposed DW-ReID with (w/) and without (w/o) image restoration on DW-DukeMTMC-reID.

Method: DW-ReID	Clear		Rain		Snow		Haze		Average	
	mAP	Rank-1	mAP	Rank-1	mAP	Rank-1	mAP	Rank-1	mAP	Rank-1
w/o restoration	80.2	89.7	73.1	88.6	74.1	85.2	56.4	75.6	70.9	84.7
w/restoration	**83.4**	**91.4**	**81.1**	**90.7**	**82.8**	**90.0**	**75.6**	**88.6**	**80.7**	**90.4**

**Table 8:** mAP and Rank-1 results of different-person ReID methods in cross-dataset evaluation by training on DW-Market-1501 while testing on DW-DukeMTMC-reID.

Methods	Clear		Rainy		Snowy		Hazy		Average	
	mAP	Rank-1	mAP	Rank-1	mAP	Rank-1	mAP	Rank-1	mAP	Rank-1
CLIP-ReID [36]	50.2	68.9	41.4	61.3	**49.7**	68.0	41.4	61.3	45.7	64.9
SVLL-ReID [37]	50.9	69.6	41.7	61.8	50.0	**68.3**	41.6	**61.4**	46.1	65.3
CLIMB-ReID [49]	30.4	45.0	28.1	42.4	30.2	44.9	23.8	38.6	28.1	42.8
**DW-ReID (Ours)**	**52.5**	**72.5**	**49.1**	**68.6**	48.9	68.2	**42.5**	60.6	**48.3**	**67.5**

**Table 9:** mAP and Rank-1 results of different-person ReID methods in cross-dataset evaluation by training on DW-DukeMTMC-reID while testing on DW-Market-1501.

Methods	Clear		Rainy		Snowy		Hazy		Average	
	mAP	Rank-1	mAP	Rank-1	mAP	Rank-1	mAP	Rank-1	mAP	Rank-1
CLIP-ReID [36]	43.4	**70.7**	41.1	67.0	38.6	59.7	33.6	58.9	39.2	39.2
SVLL-ReID [37]	**44.2**	**69.8**	41.9	**67.7**	39.1	60.8	34.5	**59.3**	39.9	**64.4**
CLIMB-ReID [49]	27.0	50.3	26.0	49.0	27.2	50.8	22.0	43.1	25.6	48.3
**DW-ReI D (Ours)**	44.1	68.1	**42.3**	67.4	**39.3**	**61.8**	**36.7**	58.7	**40.6**	64.0

## Data Availability

The datasets employed in this study are synthesized by adding weather degradation to existing publicly available datasets including Market-1501 [46] and DukeMTMC-reID [47], and the code of this work is available at: https://github.com/dvlyuying/DW-ReID (accessed on 5 February 2026).
